# A non-toxic equinatoxin-II reveals the dynamics and distribution of sphingomyelin in the cytosolic leaflet of the plasma membrane

**DOI:** 10.1038/s41598-024-67803-2

**Published:** 2024-07-23

**Authors:** Toshiki Mori, Takahiro Niki, Yasunori Uchida, Kojiro Mukai, Yoshihiko Kuchitsu, Takuma Kishimoto, Shota Sakai, Asami Makino, Toshihide Kobayashi, Hiroyuki Arai, Yasunari Yokota, Tomohiko Taguchi, Kenichi G. N. Suzuki

**Affiliations:** 1 United Graduate School of Agricultural Science, Gifu University, Gifu, Japan; 2 Department of Health Chemistry, Graduate School of Pharmaceutical Sciences, University of Tokyo, Tokyo, Japan; 3 Laboratory of Organelle Pathophysiology, Department of Integrative Life Sciences, Graduate School of Life Sciences, Tohoku University, Sendai, Japan; 4 Division of Molecular Interaction, Institute for Genetic Medicine, Hokkaido University Graduate School of Life Science, Sapporo, Hokkaido Japan; 5 Department of Biochemistry and Cell Biology, National Institute of Infectious Diseases, Tokyo, Japan; 6 Lipid Biology Laboratory, RIKEN, Wako, Saitama Japan; 7 Faculté de Pharmacie, Université de Strasbourg, Illkirch, France; 8 Department of EECE, Faculty of Engineering, Gifu University, Gifu, Japan; 9 Institute for Glyco-Core Research (iGCORE), Gifu University, Gifu, Japan; 10 Division of Advanced Bioimaging, National Cancer Center Research Institute (NCCRI), Tokyo, Japan

**Keywords:** Nontoxic equinatoxin-II variant, Sphingomyelin, Rafts, Inner leaflet, Super-resolution microscopy, Single molecule imaging, Membrane structure and assembly, Single-molecule biophysics, Phospholipids, Sphingolipids, Sterols

## Abstract

Sphingomyelin (SM) is a major sphingolipid in mammalian cells. SM is enriched in the extracellular leaflet of the plasma membrane (PM). Besides this localization, recent electron microscopic and biochemical studies suggest the presence of SM in the cytosolic leaflet of the PM. In the present study, we generated a non-toxic SM-binding variant (NT-EqtII) based on equinatoxin-II (EqtII) from the sea anemone *Actinia equina*, and examined the dynamics of SM in the cytosolic leaflet of living cell PMs. NT-EqtII with two point mutations (Leu26Ala and Pro81Ala) had essentially the same specificity and affinity to SM as wild-type EqtII. NT-EqtII expressed in the cytosol was recruited to the PM in various cell lines. Super-resolution microscopic observation revealed that NT-EqtII formed tiny domains that were significantly colocalized with cholesterol and N-terminal Lyn. Meanwhile, single molecule observation at high resolutions down to 1 ms revealed that all the examined lipid probes including NT-EqtII underwent apparent fast simple Brownian diffusion, exhibiting that SM and other lipids in the cytosolic leaflet rapidly moved in and out of domains. Thus, the novel SM-binding probe demonstrated the presence of the raft-like domain in the cytosolic leaflet of living cell PMs.

## Introduction

SM is a major sphingolipid in mammalian cells and is enriched specifically in the extracellular leaflet of the PM. Because of its high content of saturated acyl chains, SM, along with cholesterol and glycosphingolipids, is suggested to form lipid nanodomains or "lipid rafts"^1^. Lipid rafts promote the accumulation of distinct sets of proteins, including ones anchored in the extracellular leaflet by a glycosylphosphatidylinositol moiety^2^ and ones anchored in the cytosolic leaflet by lipid modification^3^. A Förster resonance energy transfer measurement in lipid bilayers containing SM demonstrated that SM formed subdomains inside of liquid-ordered domains^4^.

At present, there are a few SM-binding proteins available to visualize SM in cells. Lysenin is a SM-binding protein toxin that was isolated from the coelomic fluid of the earthworm *Eisenia foetida*^5^. Lysenin binds to SM when SM is clustered^6^. EqtII is another SM-binding protein toxin isolated from the sea anemone *Actinia equina*^7^ and preferentially binds to dispersed SM^8^. EGFP-tagged recombinant lysenin and EqtII have been used to detect SM in the PM and intracellular organelles^9^. A study with a freeze-fracture replica electron microscopy with recombinant lysenin suggested the presence of SM in the cytosolic leaflet of the PM^10,11^. This notion is further supported by a biochemical study, in which the lipid species in the extracellular or the cytosolic leaflet were quantitated^12^. Targeting the cytosolically expressed bacterial sphingomyelinase to the PM increased the levels of cellular ceramide, also suggesting the presence of SM in the cytosolic leaflet of the PM^13^.

In the present study, we generated a non-toxic variant of EqtII (NT-EqtII) so as to overcome the problem of cytotoxicity of EqtII when expressed in the cytosol. With this new probe in our hands, we could characterize the dynamics of SM in the cytosolic leaflet of the PM in living steady-state cells for the first time.

## Results

### Generation of a non-toxic EqtII variant

In order to visualize SM in the cytosolic leaflet of the living cell PM, we sought to generate the EqtII variant that lacks pore-forming activity. The pore formation by EqtII is suggested to proceed through a series of sequential processes: monomeric EqtII binding onto the surface of SM-containing membrane, its *N*-terminal helix insertion into membrane, and oligomerization to generate a final pore^14^. Given that the oligomerization of the helix appears to be critical for the cytolytic activity that is coupled with the formation of the proteinaceous pore, we attempted to generate EqtII variants with mutations in the helix or in its vicinity. Leu26 is positioned in the *N*-terminal helix^15^. By molecular dynamics simulation, Pro81 in the loop between β5 and β6 was predicted to be buried in the membrane^16^. Please note that the mutation of Val22Trp, Val8Cys/Lys69Cys, or Tyr108Iso of EqtII yielded non-toxic EqtII^17,18^ and that these variants have been used to monitor the exposure of SM in the cytosolic leaflet of organelles.

**Figure 1:**
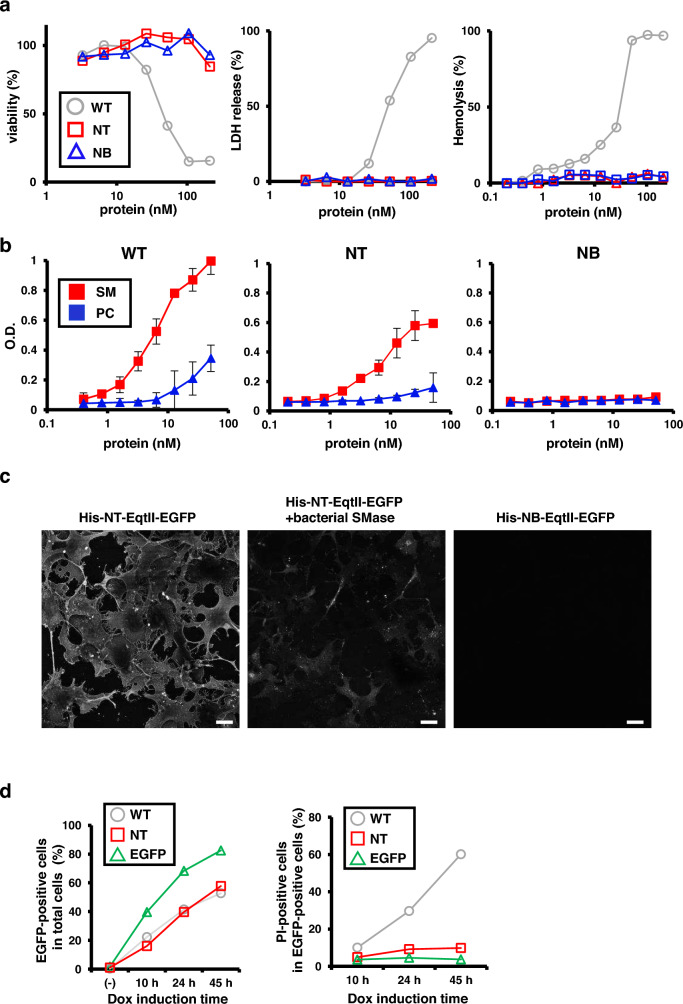
A dual mutation of EqtII abrogates its pore-forming cytotoxicity. (**a**) (Left and the middle panels) COS-1 cells were treated with recombinant EqtII proteins [WT, His-WT-EqtII-EGFP; NT, His-NT-EqtII (L26A/P81A)-EGFP; or NB, His-NB-EqtII (L26A/P81A/Y113A)-EGFP] at the indicated concentration for 30 min. Cell viability and cytotoxicity were then determined with WST-8 assay and LDH release assay, respectively. (Right panel) Mouse erythrocytes were treated with each protein at the indicated concentrations for 30 min. Hemolysis was then determined by measuring the absorbance at 600 nm. (**b**) Binding of His-EqtII proteins to SM or PC was determined by ELISA with anti-His-tag antibody. Data represent means ± standard deviations of three (WT) or two (NT and NB) experiments. (**c**) COS-1 cells were fixed and stained with 2 µg/mL His-NT-EqtII-EGFP or His-NB-EqtII-EGFP. Bacterial SMase was added to the cells 30 min before fixation. Scale bars, 20 µm. (**d**) COS-1 cells that stably express WT-EqtII-EGFP, NT-EqtII-EGFP, or EGFP in the cytosol in a doxycycline (Dox)-inducible manner were treated with Dox at 1 µg/mL. Cells were then stained with propidium iodide (PI) and analyzed with flow cytometry. Percentages of EGFP-positive cells in total cells (upper panel) and PI-positive cells in EGFP-positive cells (lower panel) are shown at 10 h, 24 h, or 45 h after Dox induction.

Fortuitously, we found that the simultaneous mutations of these two amino acid residues to Ala abolished the cytotoxicity of EqtII. Three recombinant proteins tagged with His-tag and EGFP [His-EqtII-EGFP, His-EqtII (L26A/P81A)-EGFP, and His-EqtII (L26A/P81A/Y113A)-EGFP] were expressed in *Escherichia coli* and purified. The mutation of Tyr113 to Ala was reported to diminish the SM-binding ability of EqtII^18^. We examined their cytotoxicity with three conventional assays: lactate dehydrogenase (LDH) release assay, WST-8 assay, and hemolysis assay. As shown (Fig. 1a), His-EqtII (L26A/P81A)-EGFP [referred to as NT-EqtII (non-toxic EqtII)] and His-EqtII (L26A/P81A/Y113A)-EGFP [referred to as NB-EqtII (non-lipid binding-EqtII)] did not exert the cytotoxic effect at their concentrations up to 200 nM. In contrast, His-EqtII-EGFP showed the effect on cellular viability and LDH release at 20 nM and on hemolysis at 2 nM.

Next, we examined whether NT-EqtII retained the SM-binding ability. NT-EqtII bound preferentially to SM over PC at its concentration up to 50 nM (Fig. 1b). Its affinity to SM was slightly lesser than the affinity of His-EqtII-EGFP to SM. As expected, NB-EqtII did not bind to SM^18^. The specific affinity of NT-EqtII to SM in biomembranes was confirmed by cell staining experiments. When added to the culture medium, NT-EqtII stained biomembrane of COS-1 cells, but not that of COS-1 cells pretreated with bacterial SMase (Fig. 1c). NB-EqtII did not stain biomembrane.

Lastly, we examined whether NT-EqtII could be expressed in the cytosol. We generated COS-1 cells that stably express WT-EqtII-EGFP, NT-EqtII-EGFP, or EGFP in the cytosol in a doxycycline (Dox)-inducible manner. Propidium iodide (PI) was used as a cell death indicator. After the Dox-induction, the intensities of fluorescence of PI and EGFP in individual cells were quantitated by flow cytometry. PI- and EGFP-double-positive cells were regarded as dead cells. During the Dox-induction up to 45 h, the percentage of cells positive with EGFP signal increased gradually (Fig. 1d). After 45-h induction, the percentage of PI-positive cells in cells expressing WT-EqtII-EGFP was about 60% [31.7/(31.7 + 21) × 100 = 60.2] (Supplementary Fig. S1), in line with the cytotoxic effect of WT-EqtII. In contrast, the percentage of PI-positive cells in cells expressing NT-EqtII-EGFP dropped to less than 10% [5.66/(5.66 + 52.1) × 100 = 9.8]. These results suggested that NT-EqtII could be expressed in the cytosol with less cytotoxicity.

### Single-molecule observation of NT-EqtII revealed the presence of SM in the cytosolic leaflet of the PM

**Figure 2:**
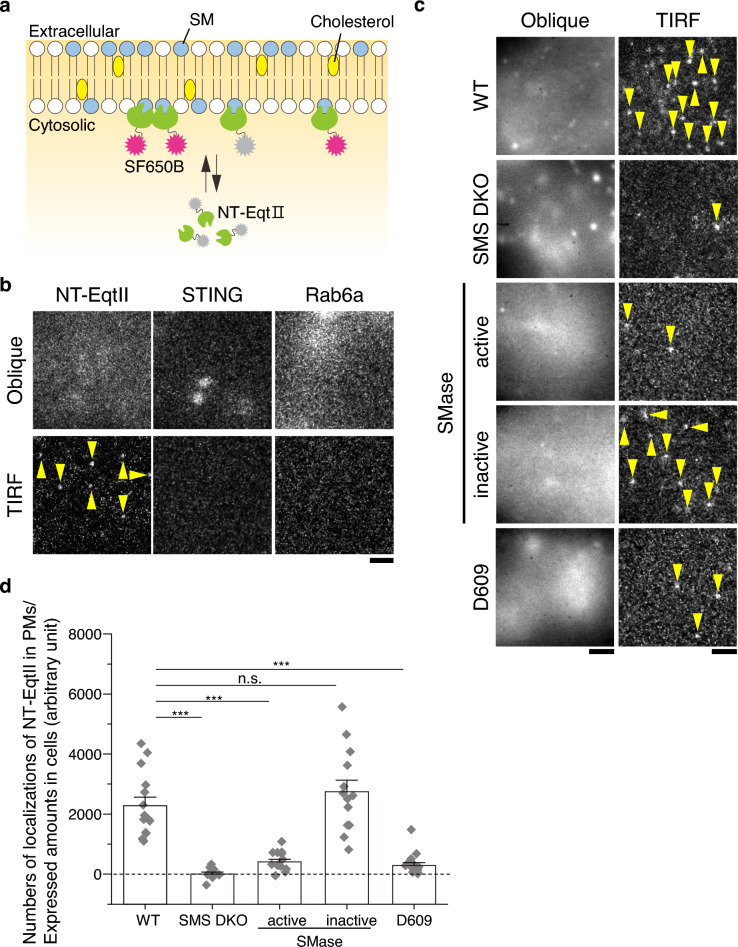
NT-EqtII exhibits a specific affinity for sphingomyelin located in the cytosolic leaflet of the living iMEF cell PM. (**a**) Schematic representation of NT-Eqt II observation in the cytosolic leaflet of the cell PM. (**b**) Fluorescent images of NT-EqtII-HaloTag7 labeled with SF650B, mEos4b-STING, and mEos4b-Rab6a in iMEFs. The images were acquired by oblique illumination (upper) and TIRF microscopy (TIRFM) (lower). Single fluorescent spots of NT-EqtII are indicated by yellow arrowheads. Scale bars, 2 μm. (**c**) Fluorescent images of NT-EqtII-HaloTag7 labeled with SF650B in various cellular contexts, encompassing wild-type (WT) iMEFs, SMS1 and 2-DKO iMEFs, iMEFs expressing WT bacterial SMase or its catalytically inactive mutant harboring Ras farnesylation sequence by which is anchored to the cytosolic leaflet of the PM, along with iMEFs subjected to D609 treatment. The images were acquired by oblique illumination (left) and TIRFM (right). Quantification of NT-EqtII-HaloTag7 expressed in cells was performed by evaluating fluorescence intensity via oblique illumination. The content of the SM probe in the cytosolic leaflet of the cell PM was quantified by measuring the numbers of single spots (yellow arrowheads) by TIRFM. Scale bars, 2 μm. (**d**) The numbers of localizations of NT-EqtII-HaloTag7 labeled with SF650B bound to the cytosolic leaflets of cell PMs, were determined by TIRFM at 4 ms/frame for 2000 frames and subsequently normalized by the expressed amounts (fluorescence intensities in the cytoplasm minus those in the background) measured by oblique illumination. The value in the vertical axis shows the difference between the numbers of localizations of NT-EqtII-Halo7-SF650B in cells expressing NT-EqtII and those in cells that do not express NT-EqtII but were incubated with SF650B. The presented error bars symbolize standard errors. *** and n.s. indicate significant (p < 0.001) and not significant differences (p > 0.05), respectively, using Welch’s *T*-test.

Next, we examined the existence of SM in the cytosolic leaflet of the PM of living immortalized mouse embryonic fibroblasts (iMEFs). NT-EqtII-HaloTag7 was transiently expressed in iMEFs, and labeled with a HaloTag®ligand SF650B. Total internal reflection microscopy (TIRFM) revealed numerous individual fluorescent spots of NT-EqtII-HaloTag7-SF650B on the PM (Fig. 2a–d). We confirmed that single-fluorescent molecules exclusively located in the Golgi (mEos4b-Rab6a) or ER (mEos4b-STING)^19^ were not observed by TIRFM at all (Fig. 2b), indicating that only single molecules in the basal PM are visible by our TIRFM system. In contrast, the number of puncta of NT-EqtII-HaloTag7-SF650B was drastically reduced in iMEFs established from sphingomyelin synthase (SMS) 1 and 2-double knockout (DKO) mice (Fig. 2c,d). For proper comparison, the number of the puncta was normalized relative to the expressed amount of NT-EqtII in cells. Furthermore, the number of the puncta was also reduced significantly by the expression of bacterial SMase tethered to the cytosolic leaflet of the PM by Ras farnesylation sequence^13^ (Fig. 2c,d). The catalytically inactive bacterial SMase mutant (D322A/H323A) did not interfere with the number of the puncta. Treatment of cells for 5 h with 200 μM D609, the inhibitor of SMS, also reduced the number of the puncta (Fig. 2c,d). These results elucidated the localization of SM in the cytosolic leaflet of the PM of living iMEFs.

**Figure 3:**
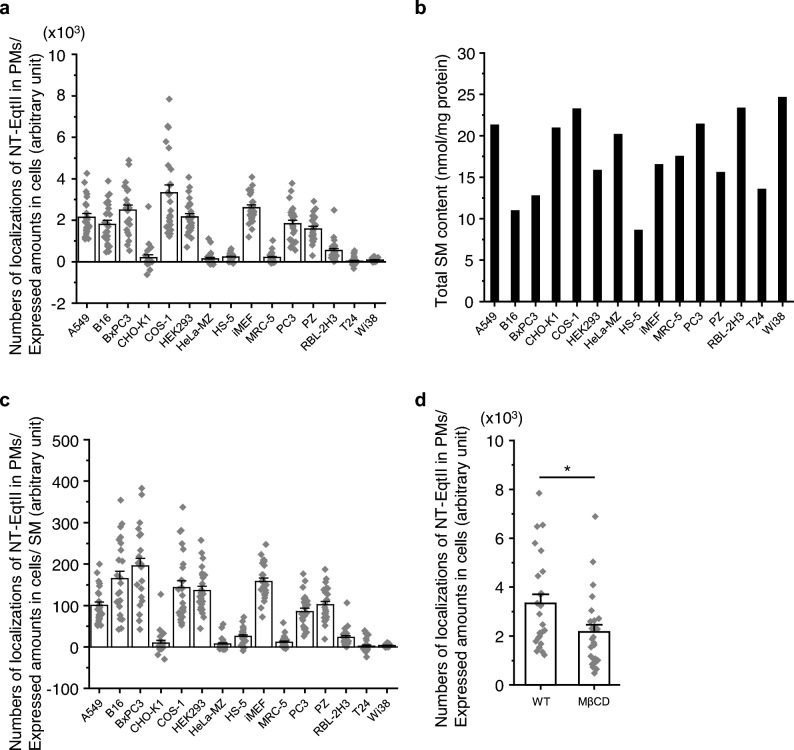
Sphingomyelin is present in the cytosolic leaflets of various cell PMs. (**a**) The numbers of localizations of NT-EqtII-HaloTag7-SF650B bound to the cytosolic leaflets of cell PMs were normalized by the expressed amounts as shown in Fig. 2d. The bars indicate standard errors. (**b**) The total SM content per 1 mg protein in each cell line was analyzed using liquid chromatography tandem-mass spectrometry. (**c**) The numbers of localizations of NT-EqtII-HaloTag7-SF650B bound to the cytosolic leaflets of cell PMs were normalized by the expressed amounts of NT-EqtII-HaloTag7 and the SM content per 1 mg protein. The bars indicate standard errors. (**d**) The numbers of localizations of NT-EqtII-HaloTag7-SF650B bound to the cytosolic leaflets of COS-1 cell PMs after cholesterol depletion with 4 mM MβCD. The error bars indicate standard errors. * indicates significant differences (p < 0.05) using Welch’s *T*-test.

We sought to determine whether the presence of SM in the cytosolic leaflet of the PM was a general feature in mammalian cells, and thus performed single-molecule observation of NT-EqtII-HaloTag7-SF650B in 14 other cell lines. Here, we used normal and cancer cells derived from various organs to determine which organ-derived cell types have more SM molecules in their inner leaflets. As shown (Fig. 3a), the numbers of NT-EqtII spots in PMs which were normalized by the expressed amounts were high in 7 cell lines (A549, B16, BxPC3, COS-1, HEK293, PC3, and PZ). The normalized number of the puncta in RBL-2H3 cells was also larger than that of the other 6 cell lines (CHO-K1, HeLa-MZ, HS-5, MRC-5, T24, and WI38). Furthermore, these numbers were further normalized by the expressed amounts of SM in cells (Fig. 3b), and the results showed that a variety of mammalian cell lines expressed SM in the cytosolic leaflet of the PM (Fig. 3c). However, we did not find any trends of the cell types that have more SM molecules in their inner leaflets. Furthermore, the amounts of SM in the inner leaflet of cell PMs seem to be irrelevant to the composition of fatty acid chains of SM expressed in cells (Supplementary Fig. S2). After cholesterol depletion with 4 mM methyl-β-cyclodextrin (MβCD), the normalized number of NT-EqtII in the PM was significantly reduced (Fig. 3d), suggesting that a PM cholesterol level was critical to the amount of SM in the inner leaflet of the PM.

### SM formed small domains that were colocalized with cholesterol and a saturated fatty acid-anchored protein

**Figure 4:**
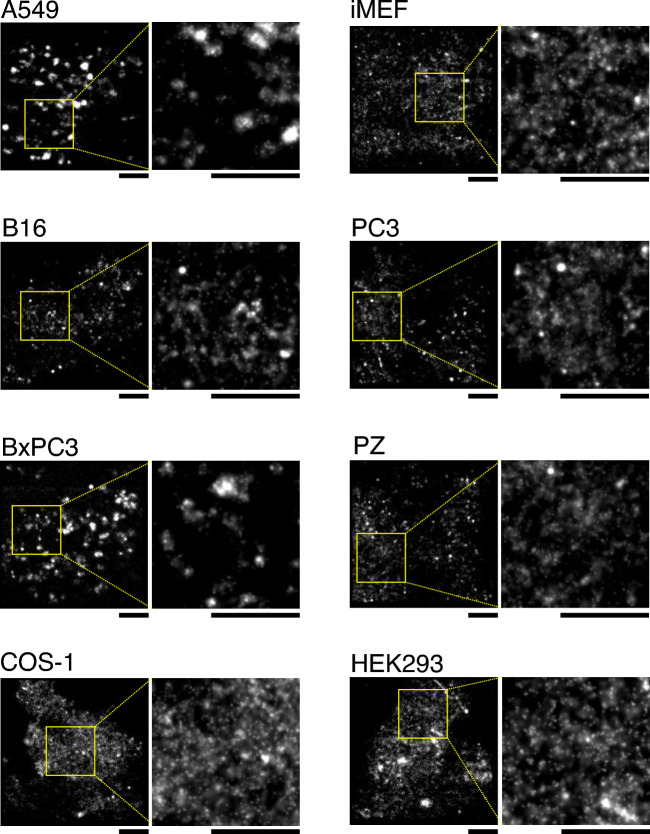
Sphingomyelin forms apparent small domains in the cytosolic leaflets of living cell PMs. The wide field (left) and enlarged (right) dSTORM images of NT-EqtII-HaloTag7 labeled with SF650B in the cytosolic leaflets of PMs of a variety of cells. The data acquisitions of dSTORM images were performed at 37 °C and at 4 ms/frame for 2000 frames: scale bars, 3 μm.

**Figure 5:**
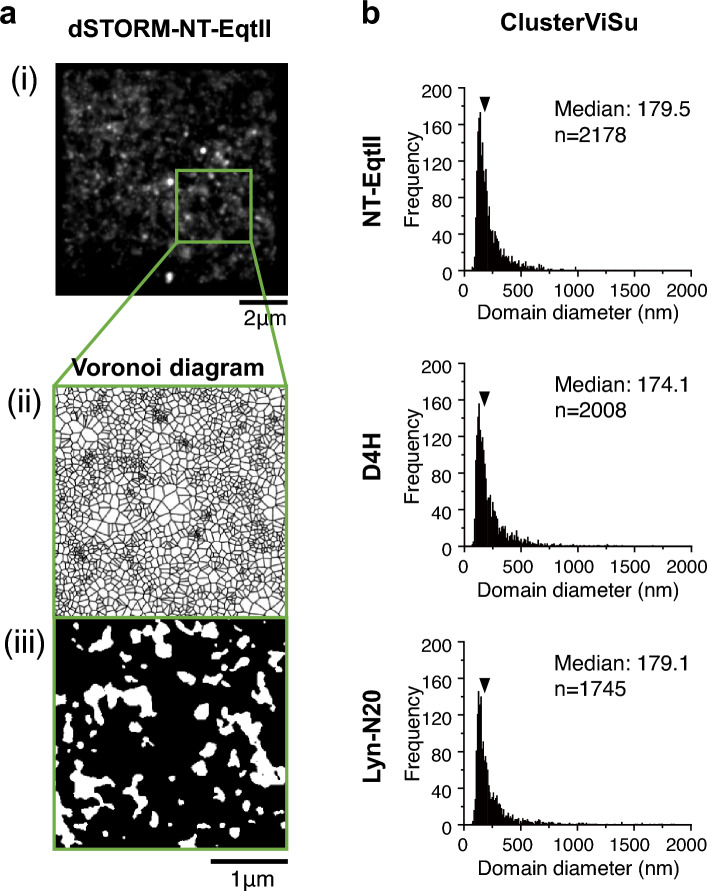
Distributions of domain areas of NT-EqtII, D4H and Lyn-N20 in the cytosolic leaflet of living COS-1 cell PMs, which were estimated by Voronoï diagram-based analysis algorithms. (**a**) A typical dSTORM image of (i) NT-EqtII-HaloTag7-SF650B expressed in the living COS-1 cell PM (data acquisition at 4 ms/frame for 2000 frames) and (ii) an example of Voronoï polygons by ClusterViSu^41^ in the square in (i). (iii) The detected domains are shown by white areas. (**b**) ClusterViSu was used for the estimation of the domain areas. The threshold was automatically determined by Monte Carlo simulation. The median of domain diameters was defined as domain sizes in this study. The domains were assumed to be a circle.

Since SM is regarded as a major component in forming lipid rafts in the extracellular leaflet of the PM, SM might form rafts also in the cytosolic leaflet of the PM. To investigate whether SM forms such domains, we performed data acquisitions of direct stochastic optical reconstruction microscopy (dSTORM) of NT-EqtII-HaloTag7-SF650B in the cytosolic leaflet of living cell PMs at 4 ms resolution for 2000 frames. As shown (Fig. 4), NT-EqtII-HaloTag7-SF650B exhibited small domains of about 150–250 nm in diameter in the cytosolic leaflet of PMs of a variety of cell lines (Fig. 5a,b, Supplementary Figs. S3 and S4). Analysis of dSTORM images in COS-1 cells (Fig. 5a) and A549 cells (Supplementary Fig. S3) by ClusterViSu detected both small numbers of large domains and large numbers of small domains. Meanwhile, few domains were observed in cell PMs where NT-EqtII-HaloTag7-SF650B was scarcely recruited (Supplementary Fig. S5).

**Figure 6:**
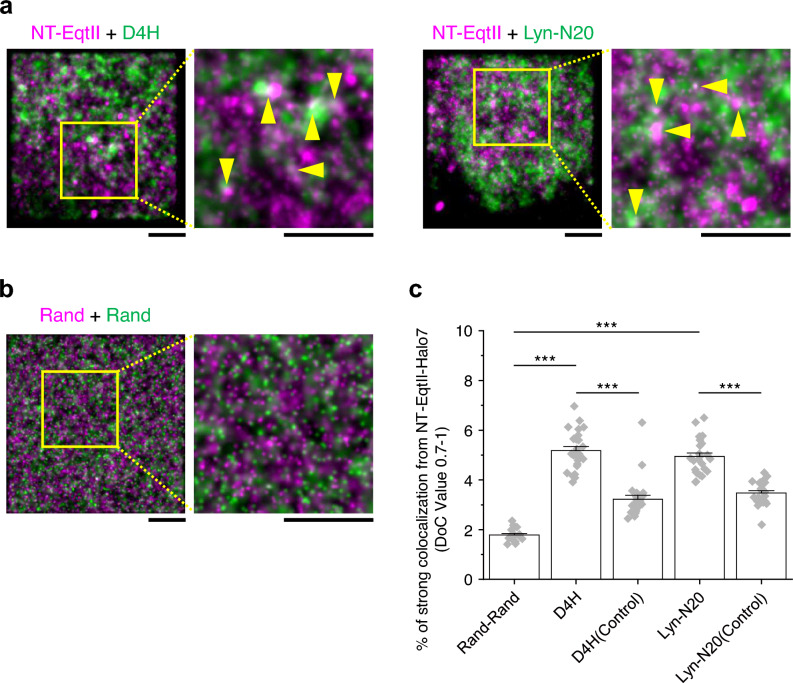
Dual-color super-resolution observation of NT-EqtII and cholesterol or Lyn-N20 and the colocalization analysis in the living COS-1 cell PM. (**a**) Dual-color super-resolution images of NT-EqtII-HaloTag7 labeled with SF650B (dSTORM, magenta) and D4H or Lyn-N20 labeled with mEos4b (PALM, green) in the inner leaflet of the living COS-1 cell PM. The data acquisitions of PALM/dSTORM images were performed at 37 °C and 4 ms/frame for 2000 frames: scale bars, 2 μm. Colocalization between the domains can be observed as indicated by yellow arrowheads. (**b**) Dual-color images of completely random distribution (Rand-Rand) generated by computer. (**c**) Degree of colocalization (DoC) scores (0.7–1.0) of NT-EqtII-HaloTag7 and mEos4b-D4H or Lyn-N20-mEos4b. Rand-Rand indicates the DoC value between computer-generated completely random distributions. The control DoC values were obtained by calculating the localization coordinates of NT-EqtII-HaloTag7-SF650B and pseudo-localization coordinates of mEos4b-D4H or Lyn-N20-mEos4b which were generated by shifting the localizations in random directions by random distances. The bars indicate standard errors. *** indicates significant differences (p < 0.001) using Welch’s T-test.

Multi-blinking of dyes for super-resolution microscopy may bring overcounting artifacts^20^, which may lead to a false perception of domain formation. The presence of molecular clusters should be verified by two-color single-molecule localization microscopy^21^. Therefore, to verify whether the lipid molecules form domains in the cytosolic leaflet, we next performed simultaneous dual-color observation of dSTORM of NT-EqtII and photoactivated localization microscopy (PALM) of D4H (a cholesterol probe)/Lyn-N20 (a representative raftophilic molecule) in live COS-1 cells. The puncta of NT-EqtII-HaloTag7-SF650B in dSTORM images appeared to be partially colocalized with those of mEos4b-D4H or Lyn-N20-mEos4b in PALM images as indicated by yellow arrowheads (Fig. 6a). Meanwhile, colocalization was not detected in the dual-color image of completely random distribution (Rand-Rand) generated by computer (Fig. 6b). The median sizes of domains of D4H and Lyn-N20 were comparable to that of NT-EqrtII (approximately 180 nm in diameter) (Fig. 5a,b).

We then analyzed the degree of colocalization (DoC)^19,22,23^ between these domains. The quantitative DoC analysis showed that the localization coordinates between NT-EqtII-HaloTag7-SF650B and mEos4b-D4H or between NT-EqtII-HaloTag7-SF650B and Lyn-N20-mEos4b were distributed in higher DoC scores (0.7–1.0) more frequently than control (Fig. 6c). The control DoC values were obtained by calculating the localization coordinates of NT-EqtII-HaloTag7-SF650B and pseudo-localization coordinates of mEos4b-D4H or Lyn-N20-mEos4b, which were generated by shifting the localizations in random directions by random distances. These results indicated that SM formed small clusters, in which raftophilic molecules such as cholesterol and Lyn-N20 were enriched, in the cytosolic leaflet of the PM in living COS-1 cells.

### Single-molecule imaging with enhanced time resolutions down to 1 ms showed that NT-EqtII underwent apparent fast simple Brownian diffusion in the cytosolic leaflet of the PM and was not confined within small domains for longer than 8 ms

We next examined whether NT-EqtII, D4H, and Lyn-N20 are temporarily confined in small domains in the cytosolic leaflet of the PM in COS-1 cells. These probes were tagged with tandem dimer (td)-StayGold^24^ and analyzed by single fluorescent-molecule imaging. The time resolution was also set at the same (4 ms) as the PALM observation.

**Figure 7:**
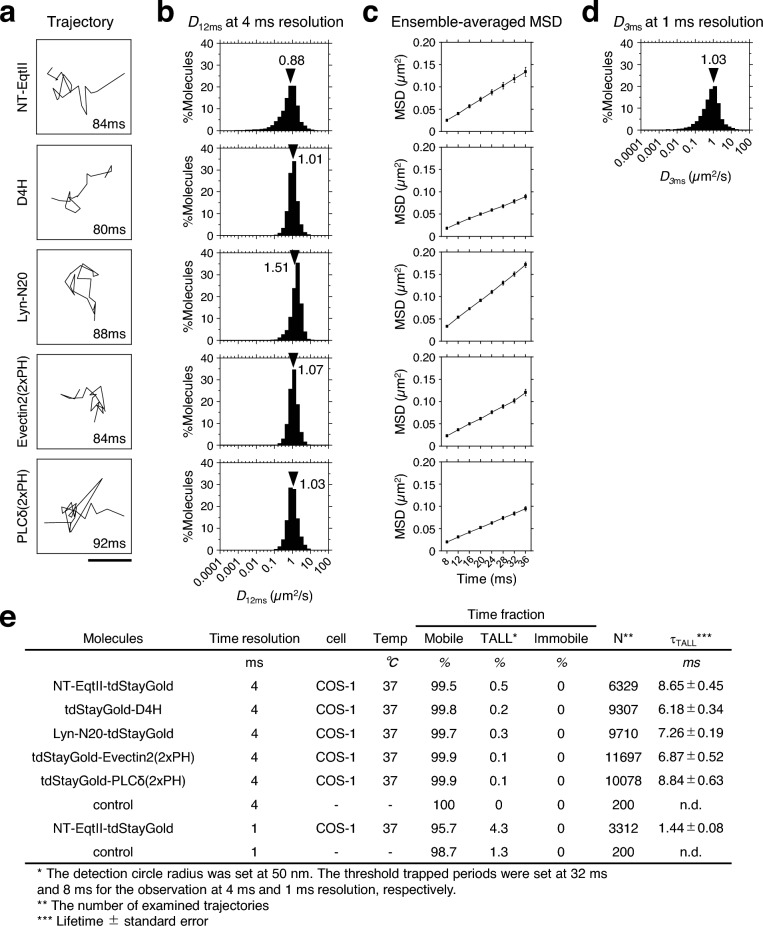
Analysis of diffusion and temporary arrest of LateraL diffusion (TALL) of single-molecules of NT-EqtII, D4H, Lyn-N20, Evectin 2 (2xPH), and PLCδ (2xPH) at 37 °C, recorded at a time resolution of 4 ms and 1 ms. (**a**) Typical trajectories of single molecules of NT-EqtII, D4H, Lyn-N20, Evectin2 (2xPH), and PLCδ (2xPH) fused with tdStayGold in the COS-1 cell PM at 37℃, exhibiting apparent fast simple Brownian diffusion. The lengths of the individual trajectories are shown at the right bottom. (**b**) Histograms showing the distributions of diffusion coefficients of single molecules shown in (**a**) in the time window of 12 ms. The black arrowheads indicate mean values. (**c**) Plots of ensemble-averaged mean-square displacements against time (MSD-Δt plots) of the single molecules shown in (**a**) at 37 °C. These plots were practically linear between 8 and 40 ms, suggesting that these molecules underwent effective simple-Brownian diffusion on this time scale. Bars indicate standard errors. (**d**) Histograms showing the distributions of diffusion coefficients of single molecules of NT-EqtII-tdStayGold in the COS-1 cell PM at 37 °C, recorded at 1 ms resolution in the time window of 3 ms. The black arrowheads indicate mean values. (**e**) The time fractions of mobile, TALL, and immobile periods, as well as TALL durations (lifetime, *τ*_TALL_) of NT-EqtII, D4H, Lyn-N20, Evectin2 (2xPH), and PLCδ (2xPH) at 37 °C, recorded at 4 ms and 1 ms resolution in the cytosolic leaflet of the intact COS-1 cell PM. These results show that SM, cholesterol, Lyn-N20, PS, and PI(4,5)P_2_ rarely exhibited TALL in the intact PM. The detection circle radius was set at 50 nm. The threshold trapped periods were set at 32 ms and 8 ms for the observation at 4 ms and 1 ms resolution, respectively. The control trajectories were obtained by computer-generated simple Brownian diffusion like coordinates.

Plots of the mean square displacement (MSD) vs. time revealed that both averages of the diffusion coefficients of the single molecules in the time window of 12 ms and the ensemble-averaged diffusion coefficients on the time scale between 8 and 40 ms were more than 0.8 μm^2^/s at 37 °C (Fig. 7a–c and Supplementary Fig. S6a). These diffusion coefficients were comparable to that of the SM probe conjugated with an organic fluorescent dye, ATTO594 via a hydrophilic nonaethyleneglycol linker (594-neg-SM)^25^, suggesting that the protein tag did not largely change the dynamics of SM. Furthermore, these diffusion coefficients were also comparable to those of a PS probe (2xPH domain of Evectin2)^26^ and a PI(4,5)P_2_ probe (2xPH domain of PLCδ) (Fig. 7 and Supplementary Fig. S6a). The statistical analysis showed that almost all these trajectories were categorized as simple Brownian diffusion (Fig. 7a–d). Furthermore, no significant entrapment/immobilization longer than 32 ms within < 100-nmΦ domains was detected with any of the lipid marker proteins at 37 °C (Fig. 7e and Supplementary Fig. S7a). Both averages of the diffusion coefficients of single molecules of NT-EqtII-tdStayGold in the time window of 3 ms and the ensemble-averaged diffusion coefficient on the time scale between 2 and 10 ms, which were obtained by observation at 1 ms resolution, were greater than 1 μm^2^/sec at 37 °C (Fig. 7d and Supplementary Fig. S6b). Furthermore, little significant entrapment/immobilization longer than 8 ms within < 100-nmΦ domains was observed (Fig. 7e and Supplementary Fig. S7b). These results suggested that, while SM formed small domains of about 180 nm (median) which colocalized with domains of cholesterol and Lyn-N20 in the cytosolic leaflet of the PM of COS-1 cells, these lipids were hardly confined in the small domains for longer than 8 ms but entered into and went out of the domains very rapidly. In other words, the presence of small domains would not retard the mobility of SM molecules, therefore, the number of SM molecules in the domains would not affect the mobility of SM molecules.

## Discussion

In the present study, we discovered the presence of SM in the cytosolic leaflet of the steady-state PM of various living cell lines by directly observing single molecules of the non-toxic SM binding probe, NT-EqtII for the first time. Furthermore, we performed single-molecule imaging of lipid-binding proteins for SM and cholesterol, and super-resolution single-molecule localization microscopy in the cytosolic leaflet of living cell PMs for the first time.

Bulk SM is synthesized on the lumenal leaflet of the Golgi by SMS1 and transported to the extracellular leaflet of the PM, whereas local SM is synthesized on the extracellular leaflet of the PM by SMS2^27,28^. Intriguingly, recent studies have identified the PM molecules involved in transbilayer movement of SM from the extracellular leaflet to the cytosolic leaflet. PMP2, a causative protein of Charcot-Marie-Tooth disease, induces the tubulation of the PM, which facilitates the flip of SM to the cytosolic leaflet^29^. TMEM16F, a PM-localized calcium-activated phospholipid scramblase, is indirectly involved in the exposure of the SM to the cytosolic leaflet of lysosomes upon lysosomal membrane damage^30^. In the present study, we found that a variety of cell lines expressed SM heterogeneously in the cytosolic leaflet of the PM. We also found that a PM cholesterol level was critical to the SM level in the cytosolic leaflet of the PM.

Simultaneous observation of PALM and dSTORM showed that SM (NT-EqtII) formed small domains that significantly colocalized with small domains of cholesterol (D4H) and Lyn-N20 in the cytosolic leaflet of the PM. On the other hand, single molecules of SM (NT-EqtII) and other lipid-binding proteins showed apparent simple Brownian diffusion in the cytosolic leaflet of the PM when they were observed at 1 and 4 ms resolutions. Furthermore, both averages of diffusion coefficients in the time windows of 12 ms (4 ms resolution) and 3 ms (1 ms resolution) (Fig. 7a–d) and ensemble-averaged diffusion coefficients on the time scale between 8 and 40 ms (4 ms resolution) and between 2 and 10 ms (1 ms resolution) (Supplementary Fig. S6a,b) were larger than 0.8 μm^2^/s. Meanwhile, previous fluorescence correlation spectroscopy observation showed that the diffusion coefficient of 594-neg-SM in the L_d_ phase of artificial giant unilamellar vesicles was larger than that in the L_o_ phase by a factor of 6–7^25^. Therefore, if SM probes temporarily reside in immobile L_o_-like domains in the cytosolic leaflet of the PM, a reduction of the diffusion coefficients and the temporal confinement should be detected. However, SM (NT-EqtII) and other lipid-binding proteins hardly underwent temporal confinement in small domains of 100 nm in diameter (Fig. 7e; Supplementary Fig. S7). These results suggested that SM, cholesterol, and Lyn-N20 rapidly diffused in the cytosolic leaflet of the PM, and entered into and went out of the SM-enriched small domains very frequently. The apparent formation of small domains detected by PALM and dSTORM observation may be due to the presence of membrane regions where those lipids are slightly more likely to stay.

**Figure 8:**
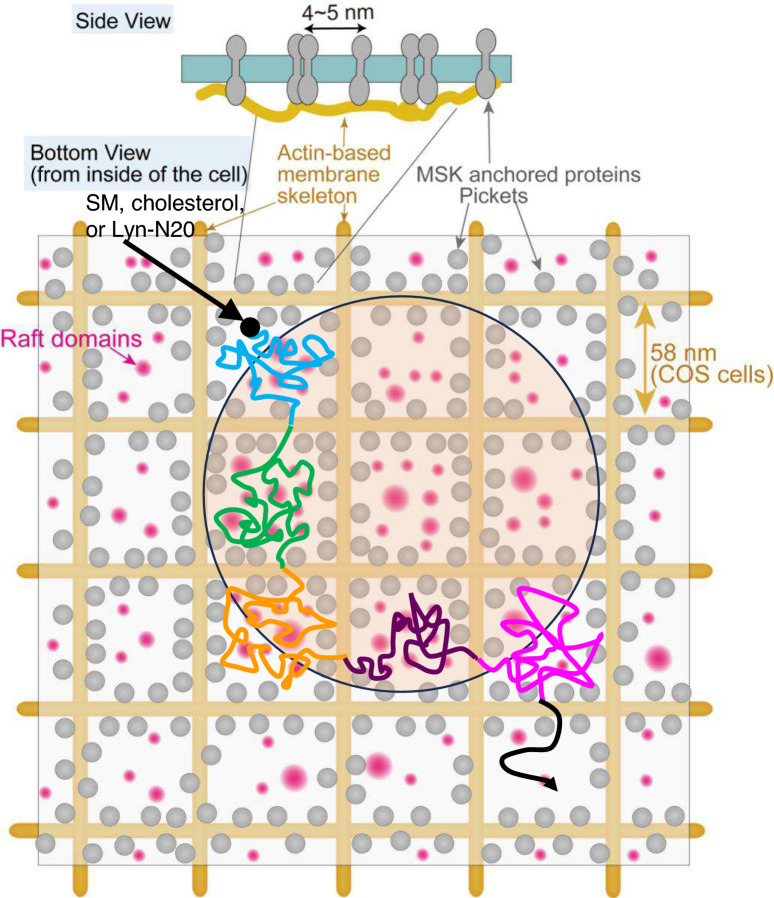
A schematic model for apparent lipid domain formation of about 180 nm in diameter, which was observed by single-molecule localization microscopy. As shown in the bottom view, the cell PM is compartmentalized by raft domains (magenta area) and the transmembrane proteins called “pickets” (gray circle) anchored to the actin-based membrane skeleton. Sphingomyelin (SM), cholesterol, and Lyn-N20 in the cytosolic leaflet of cell PMs are confined within the compartments made by transmembrane pickets due to the steric hindrance and the hydrodynamic friction effects^31,32,35^ and hop across the compartment boundary to adjacent membrane regions as indicated by the different colors in the trajectory. Since pickets exclude cholesterol, raft domains do not grow across the compartment boundary and the size is smaller than that of the compartment in the cytosolic leaflets of cell PMs. The distance between pickets is estimated to be 4–5 nm according to the Monte Carlo simulation, and the size of rafts that hop across the compartment boundary may be less than 4–5 nm. The apparent domains (beige circle) of approximately 180 nm in diameter that were visualized by single-molecule localization microscopy may consist of assemblies of smaller raft domains located within several different compartments made by pickets.

Previous studies by ultrafast (25 ~ 100 μs resolution) single-molecule observation^31–35^ have revealed that phospholipid probes exhibited short-term (1–10 ms) confined diffusion in small domains of 40–230 nm in diameter in the PMs, occasionally hopped to an adjacent domain where the phospholipids were again confined. This diffusional behavior is called “hop diffusion”, which can be observed only by ultrafast single-molecule imaging^32,36^ and not by imaging at 1 ms resolution used in this study. Meanwhile, phospholipids undergo only simple Brownian diffusion in the blebbed PM lacking cortical actin filaments even by ultrafast single-molecule observation^32,35^. Furthermore, three-dimensional reconstruction of the membrane skeleton by electron tomography^37^ revealed that the distribution of mesh size of actin-based membrane skeleton located on the PM cytoplasmic surface agreed well with the compartment size distribution determined from the value of phospholipid diffusion, using high-speed single-molecule imaging. Based on these results, it has been proposed that hop diffusion of phospholipids is induced by transmembrane proteins bound to actin-based membrane skeletons, which are called pickets^38,39^. The compartment size of phospholipids (DOPE) in COS cells that are used in this study, is reported to be 58 nm on average^40^. Since the median sizes of domains of raftophilic lipids such as SM (strictly speaking, SM may form subdomains of liquid-ordered phase as described in Introduction), cholesterol, and Lyn-N20 observed by PALM and dSTORM were estimated to be approximately 180 nm (Fig. 5a,b) by ClusterViSu method^41^, the small domains of these raftophilic lipids would contain at least four compartments formed by the “picket” anchored to the actin-based membrane skeletons. Since amino acid side chains of transmembrane pickets protrude from the α-helix, the rigid tetracyclic ring structure of cholesterol tends to be excluded from the first layer surrounding the transmembrane domain of pockets due to the steric non-conformability between them. This agrees well with the fact that most transmembrane proteins are excluded from the raft-like L_o_-phase domain^42^. Therefore, the boundary of compartments in which transmembrane picket proteins are aligned would work as the raft breaker, and raft domains may not grow across the compartment boundaries, and the size of rafts would be smaller than the compartment size. This is in line with the fact that large phase separation into L_o_-like and L_d_-like domains is observed in blebbed PMs at low (about 10 °C) temperatures, while such large phase separation have never been visualized in intact cell PMs. Nevertheless, the domains of raftophilic lipids of approximately 180 nm in diameter appeared to be formed across at least four compartments made by the transmembrane pickets anchored to the actin-based membrane skeletons (Fig. 8). These results suggest that apparent domains visualized by PALM and dSTORM may consist of assemblies of much smaller domains located within several different compartments (Φ = 58 nm) made by transmembrane pickets (Fig. 8). Since the spatial resolution of single-molecule localization microscopy is in the range of 20–30 nm^43^, it is impossible to distinguish and observe two small domains that are apart from each other at distances less than that. In the future, observation at higher spatial resolution will reveal more precise distribution and colocalization of these lipid domains.

As mentioned above, the raft-like L_o_ phase would not expand across the compartments made by transmembrane pickets which exclude cholesterol and raftophilic lipids. Monte Carlo simulations suggested that the average distance between the transmembrane pickets on the compartment boundary is 4–5 nm (Fig. 8)^34,35,44^. If SM always resides in moving rafts in the cytosolic leaflets, the rafts hop across the compartment boundaries in which pickets are aligned and need to go through a very narrow corridor (Fig. 8). If SM resides in rafts of size larger than the average distance between the pickets, both the hop frequency of the rafts and the diffusion coefficient of SM should be lower than those of non-raft molecules. However, the diffusion coefficients of SM binding protein, NT-EqtII, cholesterol binding protein, D4H and Lyn-N20 were comparable to or even larger than those of PS-binding protein (2xPH of Evectin2) and PI(4,5)P_2_ binding protein (2xPH of PLCδ) which possess one unsaturated fatty acid (Fig. 7a–c and Supplementary Fig. S6a). These results suggest that the size of the rafts that continue to hold the same SM molecules in the cytosolic leaflets would be very small, presumably less than 4 ~ 5 nm (Fig. 8). Alternatively, if SM frequently enters into and goes out of the moving rafts in the cytosolic leaflets, the size of rafts can be larger than 4–5 nm, but much smaller than the average compartment size of 58 nm. Since the average residency periods of SM, cholesterol, and Lyn-N20 in the compartment of 58 nm were estimated to be 0.85 ms according to the previous report^40^, the residency times of these raftophilic molecules in each raft would be shorter than 0.85 ms (Fig. 8).

We observed the small raftophilic domains in which SM, cholesterol, and Lyn-N20 are temporally colocalized in the cytosolic leaflet of the PM. Lyn-N20 is myristated at Gly2 and palmitoylated at Cys3^45^. Given the affinity of lipidated proteins, in particular, palmitoylated proteins to raft domains^42,46^, other lipidated proteins that localize at the PM may also have an affinity to the raftophilic domains. The function of the raftophilic domains is currently unclear. However, we envision that the raftophilic domains may serve as the signaling platform, through clustering of signaling PM proteins with lipid modifications in the cytosolic leaflet. Molecular dynamics simulation suggests that the very-long-chain (C24) acyl chain in SM interacts with acyl chains of lipids in the opposing leaflet^47^. Thus, the SM-enriched raftophilic domains in the cytosolic leaflet may be coupled with specific lipid domains in the extracellular leaflet.

In the present study, we developed a novel non-toxic SM probe that allows us to unequivocally demonstrate the presence of SM in the cytosolic leaflet of a variety of living cell PMs. This study also explicitly showed that SM forms apparent small clusters with other raftophilic lipids such as cholesterol and Lyn-N20 in the cytosolic leaflet, where these lipids frequently enter into and go out. This finding would provide a new framework for future studies on signal transduction in the cytosolic leaflet of the PM.

## Methods

### Plasmids

The plasmids encoding humanized EqtII^8^ were kindly gifted from Dr. Toshihide Kobayashi. EqtII mutants were generated by site-directed mutagenesis and then introduced into pET45b ( +) (Novagen) for bacterial protein expression and pRetroX-TetOne-puro (Clontech) for stable gene expression in COS-1 cells. The plasmids encoding bacterial SMase harboring Ras farnesylation sequence [KLNPPDESGPGCMSCKCVLS]^13^ were kindly gifted from Dr. Yusuf A. Hannun. The cDNA encoding bacterial SMase and Ras farnesylation sequence was subcloned into pEGFP-C3 vector. To generate a catalytically inactive bacterial SMase mutant, D322A/H323A mutation was introduced by site-directed mutagenesis.

To generate an expression vector for NT-EqtII-HaloTag7, the cDNA encoding NT-EqtII was cloned into pEGFP-N1 vector of which GFP was replaced by HaloTag7, the linker sequence 5’-TGGGRASGGGSGG-3’ was inserted between the NT-EqtII and HaloTag7 genes. The cDNA encoding NT-EqtII was also cloned into pPBpuro (puromycin-resistant piggyBac-vector). To generate an expression vector for mEos4b-STING, the cDNA encoding mEos4b-STING was cloned into pMX-IRES vector, the linker sequence 5’-GGGGSGGGGSGGGGS-3’ was inserted between the mEos4b and STING genes. To generate an expression vector for mEos4b-Rab6a, the cDNA encoding mEos4b-Rab6a was cloned into pMX-IRES vector, the linker sequence 5’-GGGSGGGSGGGS-3’ was inserted between the mEos4b and Rab6a genes. D4H was cloned into pET28/His6-mCherry-D4 (Clone ID: RDB_14300) in which D434S mutation was induced by site-directed mutagenesis^48^. To generate co-expression vector for NT-EqtII-HaloTag7 and mEos4b-D4H, cDNA encoding NT-EqtII-HaloTag7 and mEos4b-D4H were cloned into pEGFP-N1 vector of which GFP was deleted. mEos4b-D4H and EqtII-HaloTag7 were linked by a tandem fusion of two self-cleaving peptides (P2A and T2A), which was previously shown to have higher cleaving activity than the P2A or T2A alone^49^. To generate expression vector for Lyn-N20, the cDNA encoding Lyn-N20^50,51^ was cloned into pEGFP-N1 vector, and GFP was replaced by mEos4b. The linker sequence 5’-SGGGGSGGGGSGGGG-3’ was inserted between Lyn-N20 and mEos4b.

To generate expression vector for experiments of single-molecule observation, cDNA encoding 2xPH domain of PLCδ (a kind gift from Dr. Toshiki Itoh, Kobe University)^52^, 2xPH domain of Evectin2, Lyn-N20 or D4H was cloned into pEGFP-N1 vector, and GFP was replaced by tandem dimer (td)-StayGold (Clone ID: RDB_19609)^24^. The linker sequence 5’-GGGGSGGGGSGGGGS-3’ was inserted into the membrane molecules and tdStayGold.

### Reagents

Bacterial SMase (from Bacillus cereus, S7651) was purchased from SIGMA. Doxycycline (Dox, 631311) was purchased from Clontech. Lipids (egg SM and egg PC) were purchased from Avanti polar lipids as described^8,53^. D609 (CS-0078) was purchased from CEM.

### Cell culture

Immortalized mouse embryo fibroblast cells (iMEFs) and SMS1/2-DKO iMEFs that were kind gifts from Dr. Toshiro Okazaki^54^, COS-1 African green monkey kidney cells (American Type Culture Collection), and A549 human lung cancer cells that were a kind gift from Dr. Yasuhiko Kizuka (Gifu University), were cultured in DMEM supplemented with 10% heat-inactivated fetal bovine serum (FBS) and 1% penicillin/streptomycin/glutamine (PSG) in a 5% CO_2_ incubator. COS-1 cells that could stably express Dox-inducible EGFP, or Eqt-EGFP were established using retrovirus transfection: HEK293T cells were transfected with pRetroX-TetOne-puro constructs together with pCG-VSV-G and pCG-gag-pol and the medium containing the retroviral particles was collected. COS-1 cells were then incubated with the medium and selected with 2.5 µg/mL puromycin for a week. Chinese hamster ovary (CHO-K1) cells, PC3 human prostate cancer cells (American Type Culture Collection) were maintained in HAM’s F12 medium supplemented with 10% heat-inactivated FBS and 1% penicillin/streptomycin/glutamine (PSG. PZ human prostate cells (American Type Culture Collection) were grown in keratinocyte serum-free medium supplemented with 0.05 mg/ml bovine pituitary extract and 5 ng/ml human recombinant epidermal growth factor (Gibco). T24 human bladder cancer cells were cultured in HAM’s F12 medium supplemented with 10% heat-inactivated FBS and 1% penicillin/streptomycin/glutamine (PSG). Rat basophilic leukemia 2H3 (RBL-2H3) cells were maintained in Eagle’s minimal essential medium supplemented with 1 mM sodium pyruvate, 0.1 mM MEM non-essential amino acids, 15% FBS, and 1% penicillin/streptomycin/glutamine (PSG). MRC-5 human fetal lung fibroblast cells (American Type Culture Collection), HeLa MZ human uterus cervix cancer cells were cultured in DMEM supplemented with 10% FBS and 1% penicillin/streptomycin/glutamine (PSG). WI-38 human lung fibroblast cells (American Type Culture Collection) were cultured in MEM supplemented with 10% heat-inactivated FBS and 1% penicillin/streptomycin/glutamine (PSG). B16 mouse melanoma cells and HS-5 human marrow stromal cells (American type culture collection) were cultured in DMEM supplemented with 10% heat-inactivated FBS and 1% penicillin/streptomycin/glutamine (PSG). BxPC-3 human pancreas cancer cells were cultured in RPMI-1640 medium supplemented with 10% heat-inactivated FBS and 1% penicillin/streptomycin/glutamine (PSG).

### Plasmid transfection

Cells were transiently transfected with plasmids using Lipofectamine 2000 (Invitrogen) or Lipofectamine LTX (Invitrogen) or Lipofectamine 3000 (Invitrogen) for quantification of molecules in the inner leaflet of PMs or 4-D nucleofector (LONZA) for dual-color super-resolution microscopy and single-molecule tracking according to the manufacturer’s instructions. After transfecting the cells with cDNA plasmids, cells were seeded to a glass base dish and cultured for 36 h before the microscopic observations.

### Expression and purification of recombinant proteins

WT or mutant EqtII cDNA was subcloned into pET45b ( +) (Novagen), and the N-terminal 6xHis-tagged and C-terminal EGFP-tagged proteins were expressed in Arctic Express (DE3) RP cells (Agilent Technologies). Bacterial cultures in LB medium were induced with 0.1–0.2 mM IPTG at exponential growth phase (OD_600_ = 0.5–0.6) and grown for 24 h at 11–12 °C. The proteins were purified using a HisTrap HP column (GE Healthcare) according to the manufacturer's instructions, and then dialyzed against PBS using Vivaspin 20 (GE Healthcare).

### ELISA measurement of His-EqtII-EGFP binding to lipids

ELISA was performed as described previously^8^. In brief, 50 µL of lipid (10 mM) in ethanol was added to each well of a microtiter plate (Immulon 1; Dynatech Laboratories, Alexandria, VA, USA). After solvent evaporation at room temperature, 100 µL of 30 µg/mL bovine serum albumin (BSA) in Tris-buffered saline (TBS; 10 mM Tris–HCl, pH7.4, 150 mM NaCl) was added to each well. After washing, the wells were incubated with 50 µL each of His-EqtII-EGFP protein solution at indicated concentrations in TBS containing 10 µg/mL BSA for 1 h at room temperature. The bound proteins were detected by incubating with peroxidase-conjugated streptavidin. The intensity of the color developed with o-phenylenediamine as a substrate was measured with a Molecular Devices Spectra Max M2 (absorption at 490 nm).

### Measurement of hemolysis

Hemolytic activity was measured in mouse (C57bl/6j) erythrocytes, as previously described^53,55^. Briefly, erythrocytes were collected by centrifugation, diluted into 1 × 10^7^ cells/mL, and then the 100 µL cell suspension was mixed with each His-EqtII-EGFP protein at the indicated concentrations for 30 min. Ab600 was measured by a plate reader.

### Bacterial SMase treatment

Live COS-1 cells were treated with 1 Unit/mL bacterial SMase (*Staphylococcus aureus*) in serum-free DMEM for 60 min at 37 °C as described previously^9^.

### LDH release assay and MTT assay

Viability of COS-1 cells (1.2 × 10^4^ cells/well) was measured with MTT (3-(4,5-dimethylthial-2-yl)-2,5-diphenyltetrazalium bromide) by using Cell Counting Kit-8 (Doujin) according to the manufacturer’s protocol^53^. The release of LDH from COS-1 cells (3.2 × 10^3^ cells/well) was measured by using Cytotoxicity LDH Assay Kit-WST (Doujin) according to the manufacturer’s protocol^53^.

### Immunocytochemistry

Cells were washed with PBS briefly, fixed with 4% paraformaldehyde in PBS at room temperature for 15 min. Cells were then stained with 2 µg/mL His-EqtII-EGFP at room temperature for 1 h, washed with PBS, and then mounted in ProLong Glass Antifade Mountant (Thermo). Confocal microscopy was performed using LSM880 with Airyscan (Zeiss) with 20 × 0.8 Plan-Apochromat dry lens. Images were analysed and processed with Zeiss ZEN 2.3 SP1 FP3 (black, 64 bit) (version 14.0.21.201) and Fiji (version 2.14/1.54.f).

### Fluorescent image analysis

Quantitation of images was performed with ImageJ software (NIH).

### Propidium iodide (PI) staining and detection by flow cytometry

Expression of EGFP or EqtII-EGFP was induced in COS-1 cells after Dox-treatment for the indicated times. Cells were then collected, stained with PI for 15 min at RT, and kept on ice. Fluorescence of EGFP and PI was measured by FACS Calibur (BD Biosciences).

### Quantification of NT-EqtII in the cytosolic leaflet of the PM

The quantities of NT-EqtII-HaloTag7, labeled with SF650B and expressed in the cells were quantified by measuring the fluorescence intensities of the molecules observed by oblique illumination (Fig. 2c, left). Subsequently, the fluorescence intensities of cells in the images stacked 2000 frames were determined using ImageJ, with the subtraction of fluorescence intensity originating from the cell-free region serving as the background. Furthermore, the quantification of the NT-EqtII-HaloTag7 contents in the cytosolic leaflet of the cell PM was accomplished by measuring numbers of the single fluorescent spots, using home-built, objective-lens-type total internal reflection fluorescence microscopy (TIRFM) based on Nikon Ti2 inverted microscope (100 × 1.49 NA oil objective) equipped with a high-speed gate image intensifier (C9016-02 MLG; Hamamatsu Photonics) coupled to a sCMOS camera (ORCA-Fusion; Hamamatsu Photonics) (Fig. 2c, right). Numbers of the single spots were measured using the ThunderSTORM plugin of ImageJ with “Wavelet filtering” (B-Spline order = 4 and B-Spline scale = 4.0) and the “Local maximum method” (Peak intensity threshold = 30–50 and Connectivity = 8-neighbourhood). To eliminate the effect of non-specifically bound fluorescent molecules, numbers of the single spots in the cytosolic leaflet of PMs from cells lacking NT-EqtII-HaloTag7 expression, but treated with SF650B under the same condition, were subtracted. Subsequently, numbers of the single spots of NT-EqtII-HaloTag7 in the cytosolic leaflet of cell PMs, were normalized by the expressed quantities in the cells. This method was applied to the data in Fig. 3 as well.

### SM analysis

SM analysis were performed by the liquid chromatography tandem-mass spectrometry, LCMS-8060NX System (Shimadzu, Kyoto, Japan) as reported preiously^56^. The individual lipid contents were calculated by relating the peak area of the analyte to that of the internal standard. Data acquisition and analysis were performed using LabSolutions Insight software (Shimadzu).

### Dual-color super-resolution microscopic observation of NT-EqtII, D4H and Lyn-N20 in living cells

For live-cell dual-color PALM and dSTORM observations of NT-EqtII, D4H and Lyn-N20, COS-1 cells were sparsely seeded in a glass-base dish (4 × 10^3^ cells on the glass window of 12 mm in diameter, 0.15 mm-thick glass; Iwaki), and grown in DMEM with 10% FBS for 2 days before each experiment. Before observation, these HaloTag7-tagged molecules were labeled with SaraFluor650B (SF650B) by incubating the cells with 10 nM of the SF650B-conjugated Halo-ligand for 12 min. Single-molecules of mEos4b-D4H, Lyn-N20-mEos4b and NT-EqtII-HaloTag7-SF650B were observed using TIRFM based on a Nikon Ti2 inverted microscope (100 × 1.49 NA oil objective) equipped with two high-speed gate image intensifiers (C9016-02 MLG; Hamamatsu Photonics) coupled to two sCMOS cameras (ORCA-Fusion; Hamamatsu Photonics) as reported previously^19^. Single-molecule observation in cells was performed by illumination with an activation laser (405 nm, approximately 10.7 nW/μm^2^ for PALM observation) and excitation lasers (561 nm, approximately 28.1 μW/μm^2^ for PALM and 647 nm, approximately 55.7 μW/μm^2^ for dSTORM). In the excitation arm, a multiple-band mirror (Di01-R405/488/561/635–25 × 36, Semrock) was employed. The two-color fluorescence images of mEos4b and SF650B were separated into the two detection arms of the microscope by a dichroic mirror (Chroma: ZT561rdc-xr-UF2 or ZT647rdc-UF2). The detection arms were equipped with band-pass filters of FF01-600/37-25 (Semrock) or ET700/75 (Chroma). The data acquisitions of PALM/dSTORM were simultaneously performed at 37 °C and at 4 ms/frame for 2000 frames with an image size of 288 × 288 pixels. Each images were cropped to 120 × 120 pixels for analysis. The detection of the fluorescent spots in the images was performed by using the ThunderSTORM plugin of ImageJ with “Wavelet filtering” (B-Spline order = 4 and B-Spline scale = 4.0) and the “Local maximum method” (Peak intensity threshold = 30–50 and Connectivity = 8-neighbourhood). After spot detection, the post-processing steps of “Remove duplications” (Distance threshold = uncertainty) and “Drift correction” (cross-correlation with 5 bins) were further performed. To eliminate the effect of difference of localization densities, 15,000 localizations per image were randomly acquired for all the observations. The superimposition of images in different colors obtained by two separate cameras was performed as reported previously^50,51,57,58^. The single-molecule localization precision of mEos4b and SF650B was 26.3 nm and 22.0 nm, respectively. The final magnifications were 2 × , resulting in pixel sizes of 78 nm (square pixels).

### Estimation of domain sizes by ClusterViSu

To estimate the areas and diameters of domains of NT-EqtII, D4H and Lyn-N20 in various cell lines, we performed Voronoï-based image segmentation and cluster analysis, called ClusterViSu^41^. This method is more suitable than the SR-Tessler method^59^ in case clustered points have weak density and show a more complete detection of the cluster numbers and retrieval of their size and homogenous shape. To preserve detection accuracy, clusters containing five localizations or less were eliminated according to the previous report^41^. The median of domain diameters was defined as domain sizes in this study. The domains were assumed to be a circle.

### Quantitative analysis of the degree of colocalization between NT-EqtII-HaloTag7-SF650B and mEos4b-D4H or Lyn-N20-mEos4b

To this end, we performed degree of colocalization (DoC) analysis of PALM and dSTORM data according to the previous reports^22,23^ with modification. In brief, to estimate DoC value, for each molecule of protein A, the number of localizations of protein A (NT-EqtII-HaloTag7-SF650B) and protein B (mEos4b-D4H or Lyn-N20-mEos4b) within circles of the increasing radius was calculated, respectively, providing the density gradients of localizations of protein A and protein B around these molecules.

$$D\text{A}i,\text{A}\left(\text{r}\right)= \frac{{N}_{Ai,\text{A}}\left(\text{r}\right)}{\pi {r}^{2}} \times \frac{\pi {R}_{max}^{2}}{{N}_{Ai,\text{A}}\left({\text{R}}_{max}\right)} =\frac{{N}_{Ai,\text{A}}\left(\text{r}\right)}{{N}_{Ai,\text{A}}\left({\text{R}}_{max}\right)}\times \frac{{R}_{max}^{2}}{{r}^{2}},$$

$$D\text{A}i,\text{B}\left(\text{r}\right)= \frac{{N}_{Ai,\text{B}}\left(\text{r}\right)}{{N}_{Ai,\text{B}}\left({\text{R}}_{max}\right)} \times \frac{{R}_{max}^{2}}{{r}^{2}},$$

Here, *N*_A*i*,A_(r) is the number of localization of protein A within the distance r around protein A*i*, and *N*_A*i*,B_(r) is the number of localization of protein B within the distance r around A*i*. Then, these density gradients were corrected for the area (πr^2^), normalized by the number of localizations within the largest observed distance R_*max*_ and divided by the largest area for protein A ($\frac{{N}_{Ai,\text{A}}({\text{R}}_{max})}{\pi {R}_{max}^{2}}$) and protein B ($\frac{{N}_{Ai,\text{B}}({\text{R}}_{max})}{\pi {R}_{max}^{2}}$). Namely, the density gradients were corrected by the density at the maximum radius respectively for protein A and protein B. R_*max*_ and dR (the bin of radius for analysis) were set at 500 nm and 50 nm, respectively, and if both the number of localizations of protein A and that of protein B were less than 100 (= $\frac{{\text{R}}_{max}}{dR}$× 10) in a circle with R_*max*_, DoC values were not calculated because the density gradients cannot be accurately calculated. A uniform distribution gives an expected value of *D*(r) = 1 for all r.

The two distributions were compared by calculating a rank correlation coefficient (Spearman), in which the colocalization coefficient was weighted by a value proportional to the distance to the nearest neighbor to avoid long-distance effects^22,23^.

$$\text{S}Ai = \frac{{\sum }_{{r}_{j}=0}^{{R}_{max}}({O}_{{D}_{Ai,\text{A}}}\left({r}_{j}\right)-{\overline{O} }_{{D}_{Ai,\text{A}}})({O}_{{D}_{Ai,\text{B}}}\left({r}_{j}\right)-{\overline{O} }_{{D}_{Ai,\text{B}}})}{\sqrt{{\sum }_{{r}_{j}=0}^{{R}_{max}}{({O}_{{D}_{Ai,\text{A}}}\left({r}_{j}\right)-{\overline{O} }_{{D}_{Ai,\text{A}}})}^{2}}\sqrt{{\sum }_{{r}_{j}=0}^{{R}_{max}}{({O}_{{D}_{Ai,\text{B}}}\left({r}_{j}\right)-{\overline{O} }_{{D}_{Ai,\text{B}}})}^{2}}}$$

Here, ${O}_{{D}_{Ai,\text{A}}}\left({r}_{j}\right)$ is the rank of ${O}_{{D}_{Ai,\text{A}}}\left({r}_{j}\right)$ calculated after Spearman, and ${\overline{O} }_{{D}_{Ai,\text{A}}}$ is the arithmetic average of ${O}_{{D}_{Ai,\text{A}}}\left({r}_{j}\right)$. The colocalization value C_*Ai*_, was calculated as C_*Ai*_ = S_*Ai*_ × ${e}^{-(\frac{{E}_{Ai,\text{B}}}{{R}_{max}})}$, with E_*Ai*,B_ as the distance from *Ai* to the nearest neighbor from protein B. C_*Ai*_ (DoC score) was calculated for every single-molecule localization and ranged from − 1 (anti-colocalized or segregated), through 0 (no colocalization), to + 1 (totally colocalized). Furthermore, C_*Bi*_ was also calculated as well. The summation of C_*Ai*_ and C_*Bi*_ in the bins between 0.7 and 1 was used as an index for the colocalization. For control analysis, PALM images of mEos4b-D4H or Lyn-N20-mEos4b were replaced by pseudo-localization coordinates which were generated by shifting the localizations in random directions by random distances and overlapped with dSTORM images of NT-EqtII-HaloTag7-SF650B and the DoC scores were estimated.

### Single fluorescent-molecule tracking

For single fluorescent-molecule video imaging, COS-1 cells expressing NT-EqtII-tdStayGold, Lyn-N20-tdStayGold, tdStayGold-D4H, tdStayGold-2xPH (PLCδ), or tdStayGold-2xPH (Evectin2) were sparsely seeded in a glass-base dish (4 × 10^3^ cells on the 12-mm diameter glass window, 0.15-mm-thick glass; Iwaki), and grown for 2 days before each experiment.

Single molecules of these lipid-binding proteins or lipid-anchored proteins were observed at 37 °C at 4 ms/frame, using the Nikon Ti2 inverted microscope (100 × 1.49 NA oil objective) mentioned above as reported previously^25,57,60^. The precision of the position determinations for single stationary fluorescent tdStayGold probes was estimated to be 22.2 nm from the standard deviations of the determined coordinates of the probes fixed on coverslips.

For single-molecule observation of NT-EqtII-tdStayGold at 1 ms resolution, we used a Nikon Ti inverted microscope (100 × 1.49 NA oil objective) equipped with sCMOS cameras (ORCA-Quest; Hamamatsu Photonics) at 37 °C. The precision of the position determinations was estimated to be 17.3 nm which was determined as well.

Diffusion coefficients for individual spots were obtained as previously described^25,36^. In brief, the single-molecule mean square displacement (MSD for the time interval *Δt*, i.e. *Δr*(*Δt*)^2^) is defined as follows. For a single-molecule trajectory consisting of N determined coordinates (*x*-, *y*-positions) in a two-dimensional plane, all of the [*N*–*n* + 1] partial trajectories of *n* consecutive positions (*n* ≤ *N*) were extracted. The MSD (*N*, *n*) was then calculated by averaging the square displacements of n steps for all of these [*N*–*n* + 1] partial trajectories and, by varying *n*, the plot of MSD (Δ*t*_n_ = *nδt*) versus n*δ*t (*δt* = the duration of each image frame) was obtained. Namely, the MSD for every time interval was calculated according to the following formula:

$$\text{MSD}\left(\Delta t\text{n}\right)=\text{ MSD}\left(n\Delta t\right)=\text{ MSD}x\left(n\Delta t\right)+\text{ MSD}y\left(n\Delta t\right)$$

$$= \frac{1}{N-n+1} \sum_{j=1}^{N-n+1}\left\{{\left[x\left(j\delta t+n\delta t\right)-x(j\delta t)\right]}^{2}+\left.{\left[y\left(jdt+ndt\right)-x(jdt)\right]}^{2}\right\},\right.$$

where dt is the frame time, *x*(*jδt* + *nδt*) and *y*(*jδt* + *nδt*) describe the particle position following a time interval, *Δt*_n_ = *nδt*, after starting at position (*x*(*jδt*), *y*(*jδt*)), *N* is the total number of frames in the recording sequence, and *n* and *j* are positive integers (*n* determines the time increment). The apparent simple-Brownian diffusion was characterized by a single parameter, the “effective diffusion coefficient”. Namely, under limited conditions of the time resolution and the analysis time window, the MSD-Δt curve can be linearly fit, and only under these circumstances, the diffusion can “effectively” be described by an “effective diffusion coefficient”, given by the slope of the plot (divided by 4, by definition). The effective diffusion coefficients of a particle in the time window of 12 ms (*D*^eff^_12 ms_) and 24 ms (*D*^eff^_24 ms_) were obtained by linearly fitting its single-molecule MSD-D*t* plot at the 8 ms, 12 ms, 16 ms timepoints and at 8–40 ms timepoints (the slope divided by 4 gives the diffusion coefficient), respectively.

### Detection of temporal confinement in nanoscale domains

We attempted to detect temporary confinement/binding events or Temporal Arrest of LateraL diffusion (TALL) events, using the methods previously developed^61^, with our previous modifications^25,57^. Trajectories longer than 10 frames were used for the analysis (more than 3312 trajectories for all the molecules, with a total number of frames for each molecule greater than 33,120). The detection circle radius and the threshold residency time were set at 50 nm. The threshold trapped periods were set at 32 ms and 8 ms for the observation at 4 ms and 1 ms resolution, respectively, according to the previous work^61^. tdStayGold fixed on a glass surface was used as the standard for immobile molecules. The results obtained by this method were comparable to those previously reported^25,50,51,57,58,62^.

### Statistical analysis

Error bars displayed throughout this study represent s.d. unless otherwise indicated and were calculated from triplicate samples. Data shown are representative of three independent experiments, including microscopy images and western blotting.

### Supplementary Information

Supplementary Figures.

## Data Availability

All relevant data can be found within the article and its supplementary information.
