# Supplementary material for: A non-toxic equinatoxin-II reveals the dynamics and distribution of sphingomyelin in the cytosolic leaflet of the plasma membrane
Source: Sci Rep. 2024 Jul 23;14:16872. doi: 10.1038/s41598-024-67803-2 (PMC11266560; doi:10.1038/s41598-024-67803-2)
Supplement: Supplementary file 1 — Supplementary Figures. [file 41598_2024_67803_MOESM1_ESM.pdf]

# Supplementary Figure S1

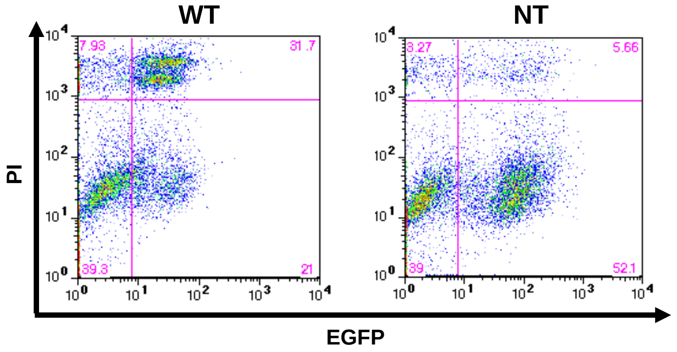


**Supplementary Figure 1| Cytotoxicity of COS-1 cells expressing WT or NT-EqtII-EGFP in the cytosol.**

COS-1 cells that stably express WT-EqtII-EGFP or NT-EqtII-EGFP in the cytosol in a doxycycline (Dox)-inducible manner were treated with Dox at 1 µg/mL for 45 h. Cells were stained with propidium iodide (PI) and analyzed with flow cytometry. The percentages of cells sorted into each section are shown at the corners.

# Supplementary Figure S2

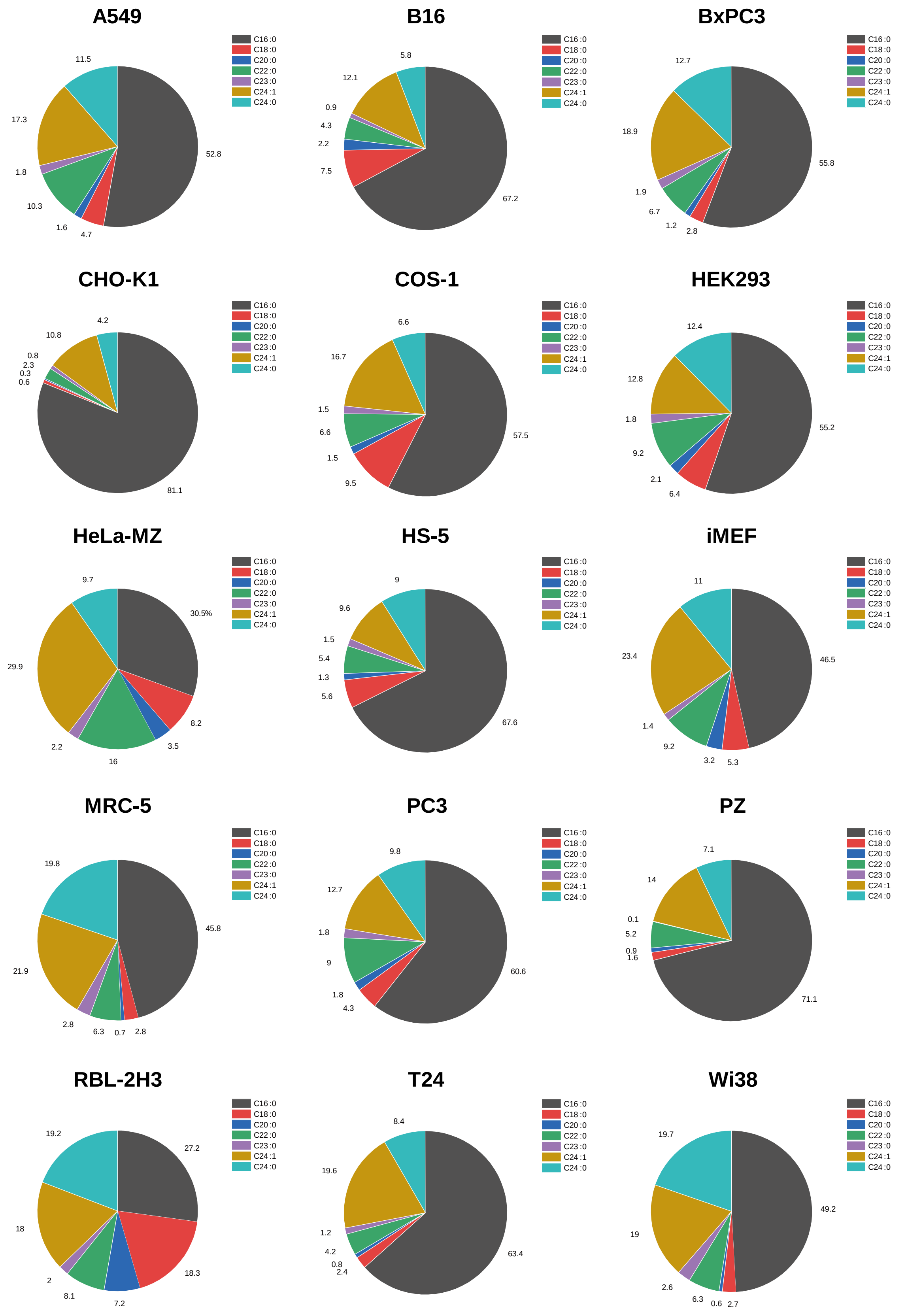


**Supplementary Figure 2| Distributions of fatty acid chains of SM in 15 types of cell lines.**
Mass spectrometry (MS) analysis was performed to estimate the ratio of fatty acid chains of SM. The values presented are average obtained from two trials.

## Supplementary Figure S3

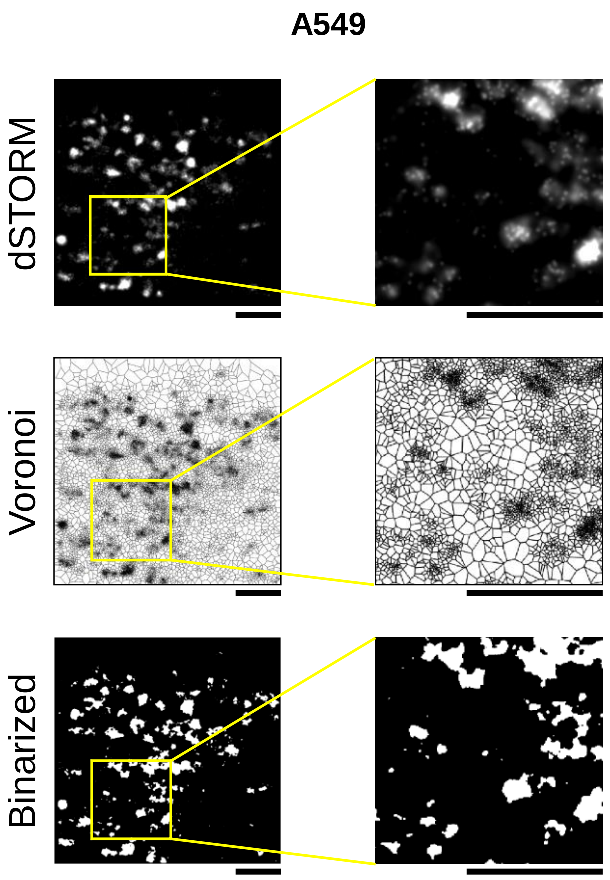


**Supplementary Figure 3| Voronoï diagram of NT-EqtII in the cytosolic leaflet of A549 cell PMs.**
ClusterViSu was used for the detection of the domain. The threshold was automatically determined by Monte Carlo simulation. The data acquisitions were performed at 37℃ and at 4 ms/frame for 2000 frames. The domains consisting of more than 6 localizations are visible in the binarized image. Scale bars, 3μm.

# Supplementary Figure S4

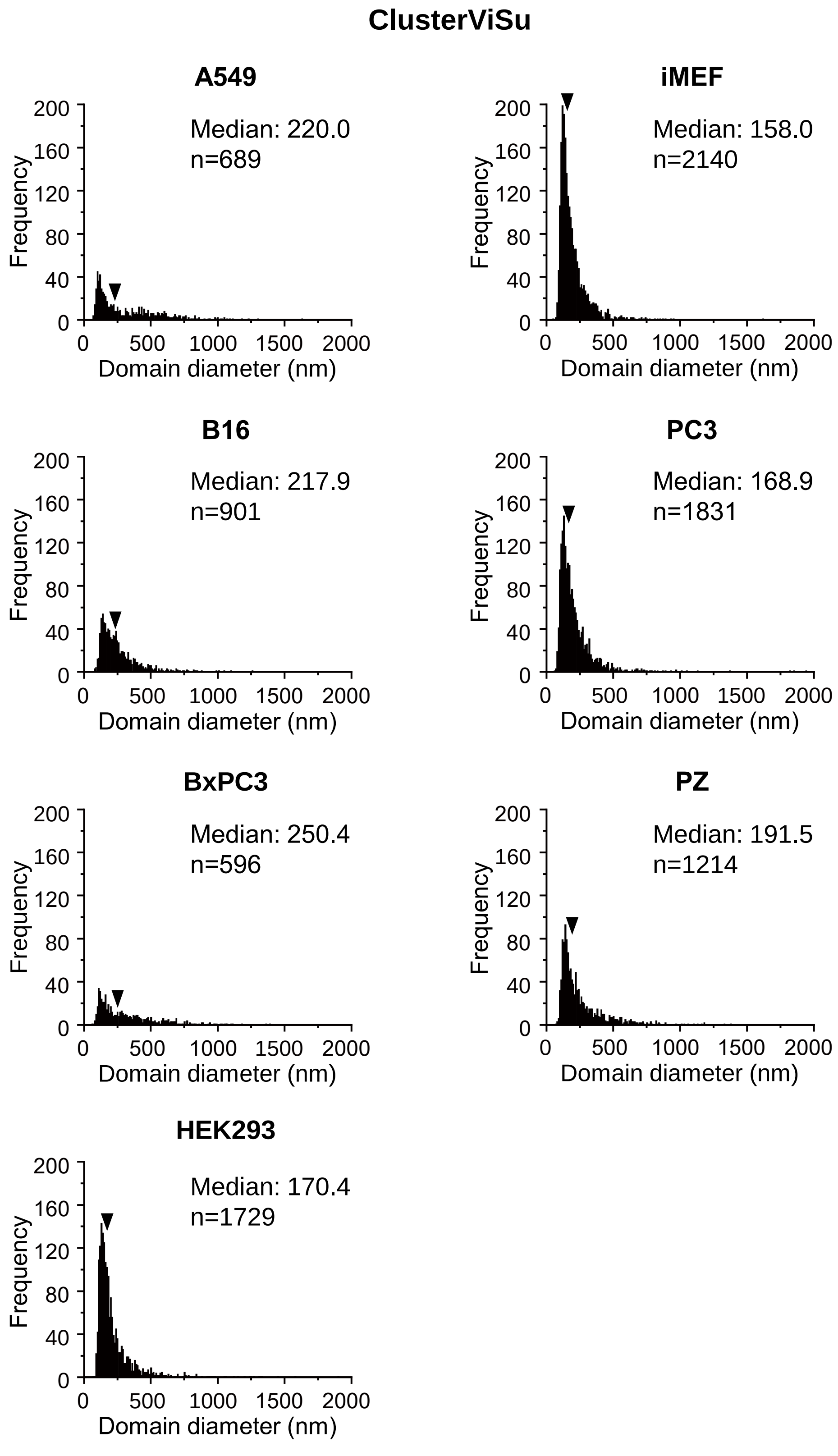


**Supplementary Figure 4| Distributions of domain diameters of NT-EqtII in the cytosolic leaflet of living cell PMs, which were estimated by voronoï diagram-based analysis algorithms.**

ClusterViSu was used for the estimation of the domain diameters. The threshold was automatically determined by Monte Carlo simulation. Black arrowheads shows the median of the domain diameter. The data acquisitions were performed at 37 ℃ and at 4 ms/frame for 2000 frames.

# Supplementary Figure S5

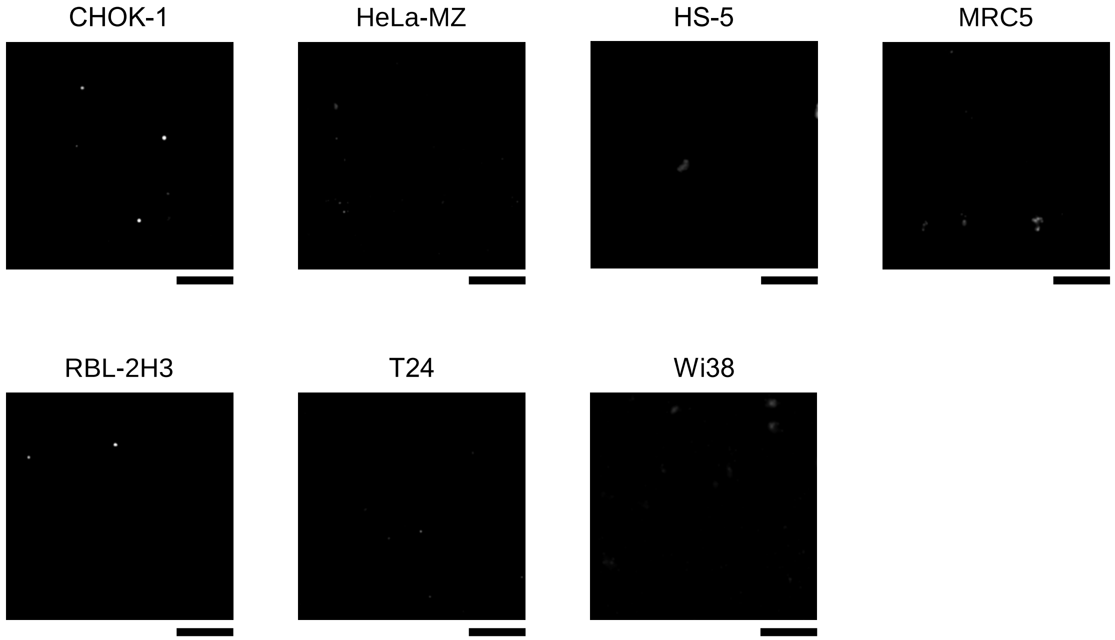


**Supplementary Figure 5| Sphingomyelin domains in the cytosolic leaflet of living cell PMs were hardly observed in cell PMs where NT-EqtII-HaloTag7-SF650B was scarcely recruited.**

The dSTORM images of NT-EqtII-HaloTag7 labeled with SF650B in the cytosolic leaflets of PMs of a variety of cells (CHOK-1, HeLa-MZ, HS-5, MRC5, RBL-2H3, T24, Wi38). The data acquisitions of dSTORM images were performed at 37 ℃ and at 4ms/frame for 2000 frames: scale bars, 3μm.

# Supplementary Figure S6

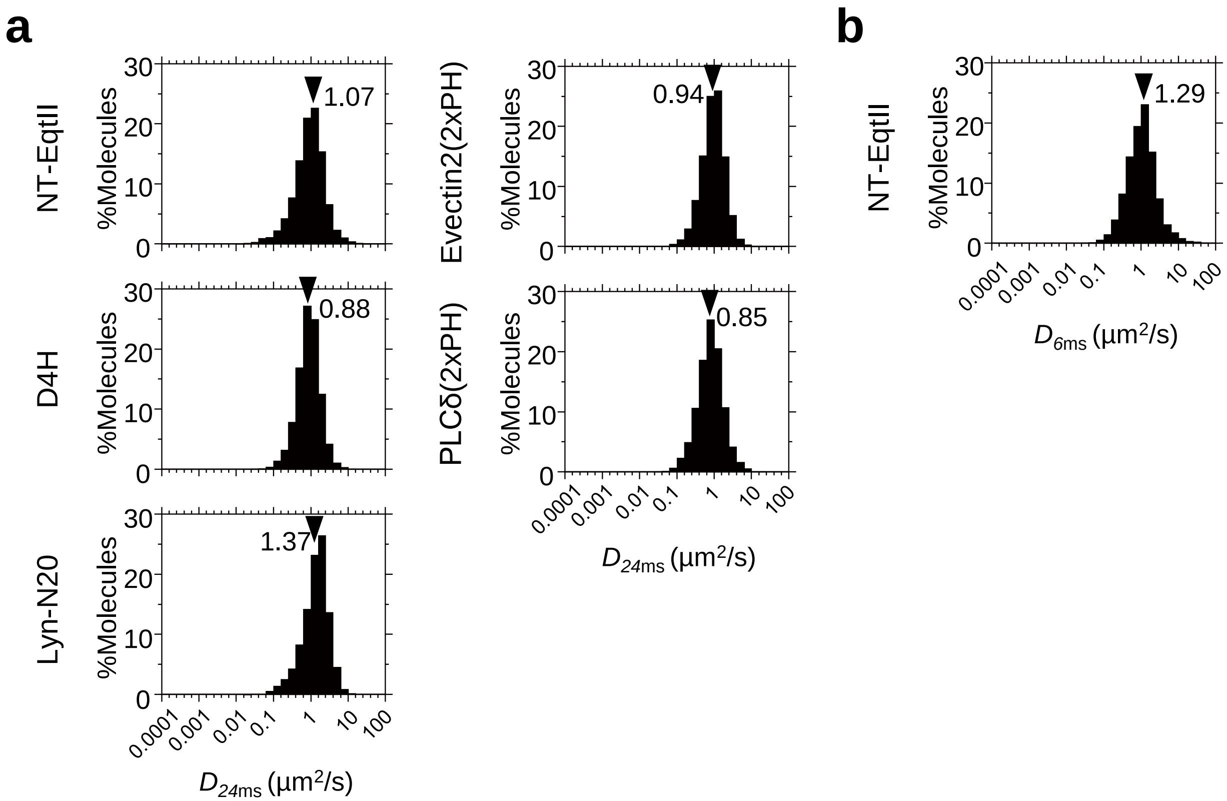


**Supplementary Figure 6| Diffusion coefficients of NT-EqtII, other molecules in the cytosolic leaflet of the living cell plasma membrane.**

(a) Single molecules of NT-EqtII, D4H, Lyn-N20, Evectin2(2xPH) and PLCδ(2xPH) tagged with (td)-StayGold in the cytosolic leaflet of the COS-1 cell plasma membranes were observed at 4-ms resolution and 37 ˚C. Diffusion coefficients ($D_{24ms}$) were evaluated from the slope of plots of mean square displacements against time (MSD-$\Delta$t plots) between 8 and 40 ms. Black arrowheads indicate the mean values of $D_{24\ ms}$.

(b) Diffusion coefficients ($D_{6ms}$) of single molecules of NT-EqtII tagged with (td)-StayGold observed at 1-ms resolution were evaluated from the slope of plots of mean square displacements against time (MSD-$\Delta$t plots) between 2 and 10 ms. Black arrowhead indicates the mean value.

# Supplementary Figure S7

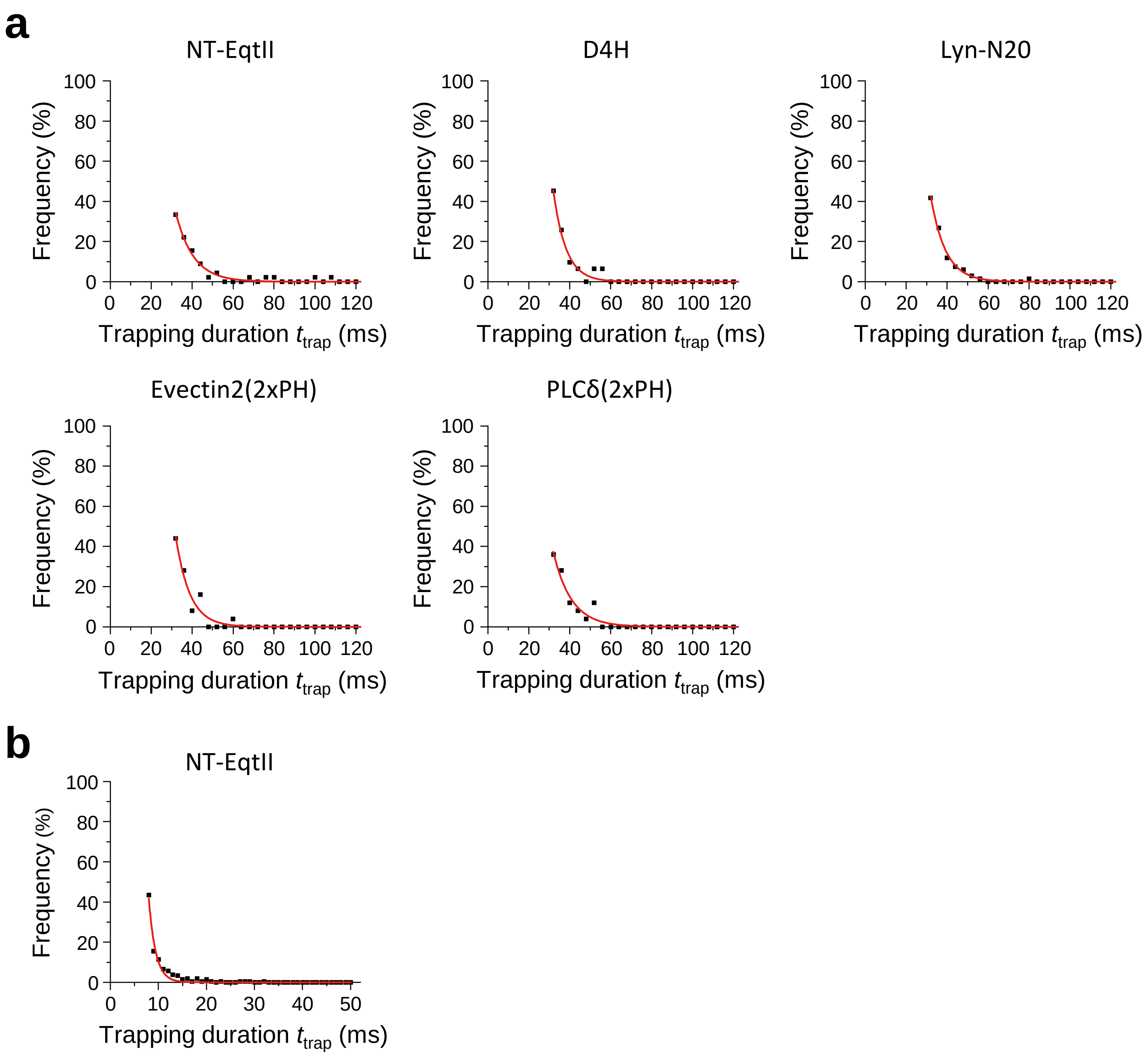


**Supplementary Figure 7| Temporary arrest of LateraL diffusion (TALL) analysis of NT-EqtII, D4H, Lyn-N20, Evectin 2 (2xPH), and PLCδ (2xPH) at 37 ˚C, recorded at 4-ms and 1-ms resolution**

Histograms showing the distributions of TALL periods fitted with an exponential decay curve at 4 ms resolution (a) and 1 ms resolution (b).
